# Hydroxytyrosol Enrichment of Olive Leaf Extracts via Membrane Separation Processes

**DOI:** 10.3390/membranes12111027

**Published:** 2022-10-22

**Authors:** Costas S. Papageorgiou, Stathis Lymberopoulos, Panagiotis Bakas, Dimitris P. Zagklis, Varvara Sygouni, Christakis A. Paraskeva

**Affiliations:** 1 Laboratory of Transport Phenomena and Physicochemical Hydrodynamics (LTPPH), Department of Chemical Engineering, University of Patras, GR26504 Patras, Greece; 2 Institute of Chemical Engineering Sciences, FORTH/ICE-HT, GR26504 Patras, Greece

**Keywords:** olive leaves, hydroxytyrosol, oleuropein, membrane filtration, ultrafiltration, nanofiltration

## Abstract

Antioxidants isolated from plant materials, such as phenolics, have attracted a lot of attention because of their potential uses. This contributes to the idea of the biorefinery, which is a way to produce useful products from biomass waste. *Olea europaea* byproducts have been extensively investigated for their large contents in phenolics. Oleuropein is a phenolic compound abundant in olive leaves, with its molecule containing hydroxytyrosol, elenolic acid, and glucose. In this work, olive leaf extracts were treated using different combinations of ultrafiltration and nanofiltration membranes to assess their capacity of facilitating the production of hydroxytyrosol-enriched solutions, either by separating the initially extracted oleuropein or by separating the hydroxytyrosol produced after a hydrolysis step. The best performance was observed when an ultrafiltration membrane (UP010, 10,000 Da) was followed by a nanofiltration membrane (TS40, 200–300 Da) for the treatment of the hydrolyzed extract, increasing the purity of the final product from 25% *w/w* of the total extracted compounds being hydroxytyrosol when membrane processes were not used to 68% *w*/*w*.

## 1. Introduction

In recent years, secondary metabolites from plant materials such as phenolics have attracted a lot of attention because of their potential to be used as dietary supplements [1,2,3], food additives [4,5], packaging additives [6,7], and to protect from natural oxidation and sun exposure. This attention is well justified if one considers the numerous sources from which these substances can be obtained. The majority can be found in fresh food, but there are also great quantities of these compounds in plant waste and byproducts from the food sector [8,9,10]. Phenolics can be extracted using various techniques (solvent extraction, supercritical extraction, Soxhlet extraction, etc.) and can be purified to be added in various other products such as oils, juices, yogurts, supplements, cosmetics, and drugs. This option contributes to the idea of the biorefinery [11,12], which is a way to recover useful resources from plant waste (biomass). The main components of biomass are cellulose, hemicellulose, and lignin, but it also contains extractives, which are various molecules connected to plant metabolism (proteins, lipids, sugars, phenolics, etc.). Phenolics, usually, exhibit protective functions against natural factors, insects, and pathogens. They can be found in great quantities in wild cultivars that are characterized by better defensive mechanisms than their commercial counterparts. Their concentrations can change seasonally and can be influenced by external factors that may induce stress to the plants. Not all phenols have useful functions, but there is a great number of them with antioxidant capacities and medicinal uses that even resemble some licensed drugs [13]. Their bioavailability is most of the time in question and a lot of research is going into coupling them with useful agents (e.g., encapsulation) or altering their chemical structure (e.g., acetylation) to make them more easily absorbed and utilized by the human body. An important advantage is that these compounds already exist in feedstocks and can be obtained cheaply or even for free, and the only cost is related to material processing [14,15]. This, however, does not mean that it is always easy to procure the necessary biomass or to safely transport and/or store it.

Olive trees (*Olea europaea*) have been extensively examined for their large content of phenolics, which have been shown to have a high antioxidant capacity and beneficial effects on human health such as anti-inflammatory, cardioprotective, and neuroprotective ones [16,17]. The most abundant and studied phenolic compound in olive trees is oleuropein [18,19], a glycosylated secoiridoid that gives olives their characteristic bitter taste. This molecule is a combination of three other molecules, namely hydroxytyrosol, elenolic acid, and glucose, and contains an ester and glycosidic bond that can be hydrolyzed to obtain the three molecules separately. Hydroxytyrosol is another potent antioxidant that has attracted a lot of research interest [20,21,22]. Oleuropein owes its properties mainly to hydroxytyrosol, which contains the drastic catechol group. Hydroxytyrosol is a smaller molecule, therefore its activity-to-weight ratio is larger than oleuropein and it does not have the same bitterness, which is attributed to elenolic acid, making it more attractive as a product. Hydroxytyrosol can be found in solid and water residues of olive mills [23,24,25] and is found hydrolyzed mainly because oleuropein is subjected to enzymatic hydrolysis when olives are crushed. However, when olive leaves are extracted, oleuropein is not hydrolyzed and therefore a hydrolysis step must be included. Hydroxytyrosol may also be synthesized chemically [26,27]. However, its chemical synthesis is convenient because it does not have the same number of impurities compared with hydroxytyrosol obtained from wastes and, although greater product purities may be obtained, the costs of the reaction step and the precursors may significantly increase the product price.

The present study builds on a previously published study regarding the extraction of phenols from olive leaves [28]. In the previous study, oleuropein was extracted from dry olive leaves and was hydrolyzed to obtain hydroxytyrosol, using liquid–liquid extraction [28]. Carefully tuning the pH value of the aqueous phase before liquid–liquid extraction allowed to obtain high purities of hydroxytyrosol (>40%) without compromising the yield of the extracted compound (>10 g hydroxytyrosol per g of dry leaves). In this study, an improvement on the proposed process is examined, namely including a membrane separation step. Membrane separation has been proposed as a way to purify wastewaters and also to fractionate the waste into substances that can be utilized [29,30,31,32,33,34,35,36,37]. The idea of “molecular sieving” was applied with two goals in mind, being tested separately in different experiments. Firstly, it was tried to separate oleuropein from larger molecules before hydrolysis to reduce unwanted reactions and complications from impurities. Secondly, it was tried to first hydrolyze oleuropein and then use very “tight” nanofiltration (NF) membranes to separate this small molecule from larger ones before liquid–liquid hydrolysis. Small molecules in the hydrolysate usually include small phenolics and monosaccharides. Separating these two categories would make it easier for hydroxytyrosol to be separated with liquid–liquid extraction because polar molecules such as sugars would end up in the aqueous phase and phenolics with small pK_a_ would also end up in the aqueous phase with proper pH tuning, thus further increasing the purity of hydroxytyrosol in the final product.

The aim of this study was to demonstrate the effect of different membrane filtration schemes on the recovery of hydroxytyrosol-enriched solutions from olive leaf extracts. Apart from the different UF and NF membranes employed, the step where membrane filtration was implemented (before or after oleuropein hydrolysis) was also examined.

## 2. Materials and Methods

### 2.1. Pretreatment

The olive leaves used in this study were collected during the summer of 2020 from the olive orchard of the University of Patras, Achaia, Greece. Mostly young leaves were collected from new growth as these were easily removed and served as pruning for the trees. The leaves were washed with tap water to remove dirt and other non-plant matter and then placed in a room with good air circulation to remove as much humidity as possible over the course of two weeks. When the leaves were dry, the relative humidity was measured and found to be approximately 5%, which was enough for complete pulverization with a typical blade grinder. The olive powder was then sieved to particles smaller than 0.71 mm and stored in an airtight container at −5 °C for future experiments. 

### 2.2. Physicochemical Processes

Solid–liquid extraction was performed in a jacketed vessel under magnetic stirring to control the temperature in the extraction medium. A heating bath provided a constant flow of hot water in the jacketed vessel and the olive leaf powder was introduced when the liquid’s temperature reached 40 °C. The extraction lasted for 20 min according to previously published results [28], as these extraction parameters have been proven to be more than adequate for the recovery of most of the extractives. A solution of 70% *v*/*v* ethanol in water was used as solvent. After extraction, the solid powder was separated from the liquid via vacuum filtration with 0.22 μm hydrophilic PVDF filters (Membrane Solutions, Auburn, AL, USA) and most of the ethanol was evaporated with vacuum evaporation. This step was required to conserve sensitive substances and to avoid ethanol’s interference with the membrane separation and liquid–liquid extraction further down the process. The membrane separation step was tested at two different points of the process, during different experiments, to determine the best way to utilize it. In the first case, membranes were used right after solid–liquid extraction aiming to separate oleuropein from larger molecules. In the second case, first, hydrolysis took place to break the bonds of oleuropein and then, membranes were used to separate hydroxytyrosol from larger molecules.

Acid hydrolysis was vital to produce hydroxytyrosol because in its natural state in the olive leaves it is “locked” in the molecule of oleuropein. Under acidic conditions, it is relatively easy for oleuropein’s bonds to break. Sulfuric acid was the catalyst of choice because it can be removed as gypsum with solid–liquid separation when the hydrolysate is neutralized with Ca(OH)_2_. Hydrolysis took place by submerging the reaction vessel in a boiling bath for 20 min. After that time, the vessel was removed from the boiling bath and it was cooled to room temperature. Next, the basic solution was introduced slowly while monitoring solution’s pH. The pH value was set at ~8, the solution was filtered again for solid particle removal, and the treated hydrolysate was brought to the next process step.

The process was finalized with a liquid–liquid extraction using ethyl acetate. At this step, setting the pH value close but less than the pK_a_ of hydroxytyrosol was very important for raising the concentration of hydroxytyrosol in the extract without compromising its recovered amount [28]. The hydrolysate was extracted three times with ethyl acetate, with a total volume of the organic phase equal to the volume of the aqueous phase. After extraction, the three portions of the organic phase were mixed and brought into liquid–liquid contact with a brine solution (sat. NaCl) to remove any residual water and impurities from the organic phase. The brine was discarded and the ethyl acetate solution was greatly condensed in a rotary evaporator. After that, the solution was left in small open vessels for 1 to 2 days in a fume hood for the rest of the solvent to evaporate and to retrieve the final rich-in-hydroxytyrosol extract.

**Figure 1:**
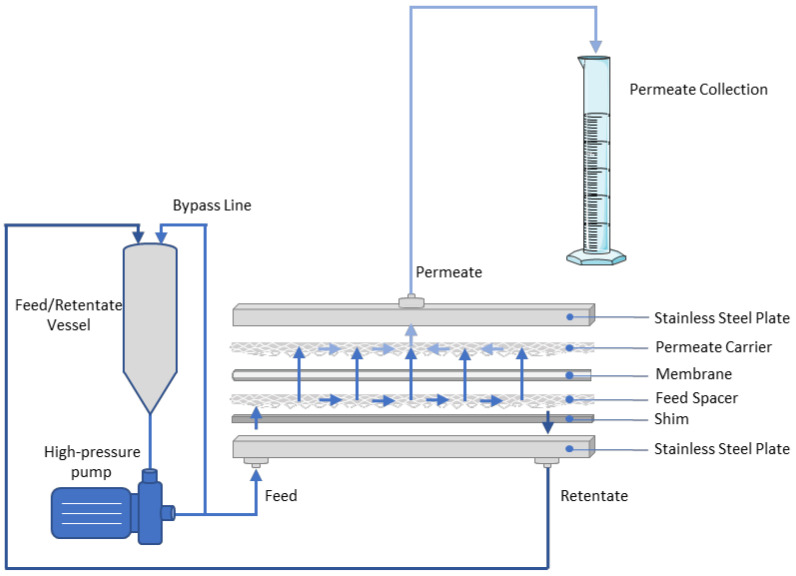
Membrane separation unit that uses flat-sheet membranes and operates in batch, cross-flow mode, and flat-sheet unit loadout. All parts (permeate carrier, membrane, feed spacer, shim) were carefully put on top of each other and then pressed with the outer steel elements. Steel elements were held together with a hydraulic press.

A membrane separation unit operating in batch, cross-flow mode, and using flat-sheet membranes was set up to test the various membrane schemes on a laboratory scale. As can be seen in Figure 1, the aqueous solution was fed into a cylindrical vessel with its outlet leading to a plunger pump that was suitable for all the different membranes examined, from high-flux ultrafiltration (UF) flat sheets to “tight” NF ones. Downstream of the pump, a bypass line with a choking valve was used to slowly drive the pumping liquid through the membrane module. The process retentate was recirculated in the feed tank, while the permeate stream was collected in a separate vessel. Due to friction, an increase in recirculated feed stream temperature was observed, but it was kept below 35 °C using a water bath. After membrane filtration was initiated, the valve in the retentate line was slowly closed to restrict the flow in the outlet and to create the appropriate pressure on one side of the membrane (depending on the membrane type) and to create a flux through the membrane. When the appropriate pressure was reached, the permeate was collected. The pressure was continuously monitored during filtration. As the feed stream became more concentrated, the transmembrane pressure increased and therefore appropriate adjustments to the valve were made to keep the pressure constant. Especially in the initial treatments with the UF, fouling and concentration polarization effects were intense and significantly reduced the permeate flux (Appendix A). The flat-sheet membranes were acquired from Mann+Hummel (Ludwigsburg, Germany) and were the following: UP010, NP010, NP030, US100, UA60, TS50, TS40, SB90, X-20, XN45. Their characteristics are summarized in Table 1. The loading parts in the flat-sheet membrane module are shown in Figure 1.

The experiments were specifically designed to determine the best process step to include membrane separation and the number and type of consequent membranes to be used. 

### 2.3. Analyses

Two types of analyses were performed on the samples received after the final treatment step and on the different liquids that were treated with membranes (feeds, permeates, and retentates). The first type of analysis was using photometric methods (Folin–Ciocalteu reagent assay and L-tryptophan reagent for dissolved sugars) and the second was a chromatographic method to quantify hydroxytyrosol. The Folin–Ciocalteu assay [38] was measured in gallic acid equivalents (mg/L). However, this method does not quantify accurately the sum of the phenolic compounds but rather provides a meter of antioxidants in the sample by measuring the reductive capacity of the substances in it. Since it is a relatively easy and inexpensive assay, it was chosen in this case as a general gauge for the amounts of phenolics in the samples. The dissolved sugars’ assay [39] depends on the mild reducing properties of sugars, so it is not an accurate quantification method but is useful as an indicator of their general assessment. Data are presented as grams of standard equivalents per kilogram of dry olive leaf powder, by dividing the method’s signal (g/L) by the solid/liquid ratio (kg/L) during extraction. Again, this is not an accurate quantification indicator, but it provides the order of magnitude of phenolics and sugars.

The second type of analysis that was carried out was chromatographic. The chromatographic method was vital to track the concentration of hydroxytyrosol at the end of the process and was performed on a Waters Alliance 2695 HPLC. (Waters, Milford, MA, USA) The column that was used was a reversed phase C-18 column (Phenomenex, Torrance, CA, USA, Prodigy, 5 μm, ODS-3 100 Å, 100 × 4.6 mm). The mobile phase was 0.1% trifluoroacetic acid (TFA) in HPLC-MS grade water (A) and HPLC grade acetonitrile (B). The hydroxytyrosol standard was purchased from Sigma Aldrich with a purity greater than 98%. The method used gradient elution, starting at 10% acetonitrile, constant for 10 min, and then gradually increased to 90% acetonitrile over the course of 30 min. The hydroxytyrosol peak was observed at approximately 8.5 min.

All experiments were carried out in duplicate and each sample was measured in duplicate as well. The results are presented as mean values, with their standard deviation as error bars. Finally, statistical analysis of the results was carried out using the Student’s ^t^-test, with *p* values lower than 0.05 considered as statistically significant. For the statistical analysis, Microsoft Excel was used.

## 3. Results and Discussion

**Figure A1:**
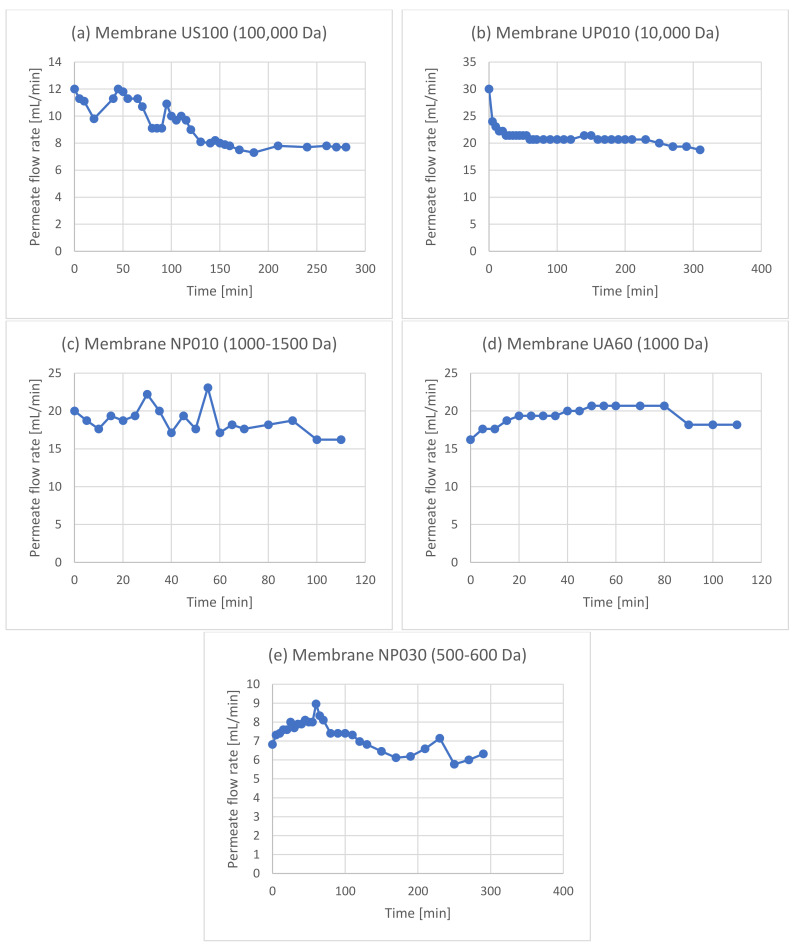
Permeate flow rates observed in the membrane treatment scheme after solid–liquid extraction in which two ultrafiltration steps ((**a**) US100, (**b**) UP010) were used in line and the permeate from the 10,000 Da membrane was split into three equal parts and was treated with three different nanofiltration membranes ((**c**) NP010, (**d**) UA60, (**e**) NP030).

**Figure A2:**
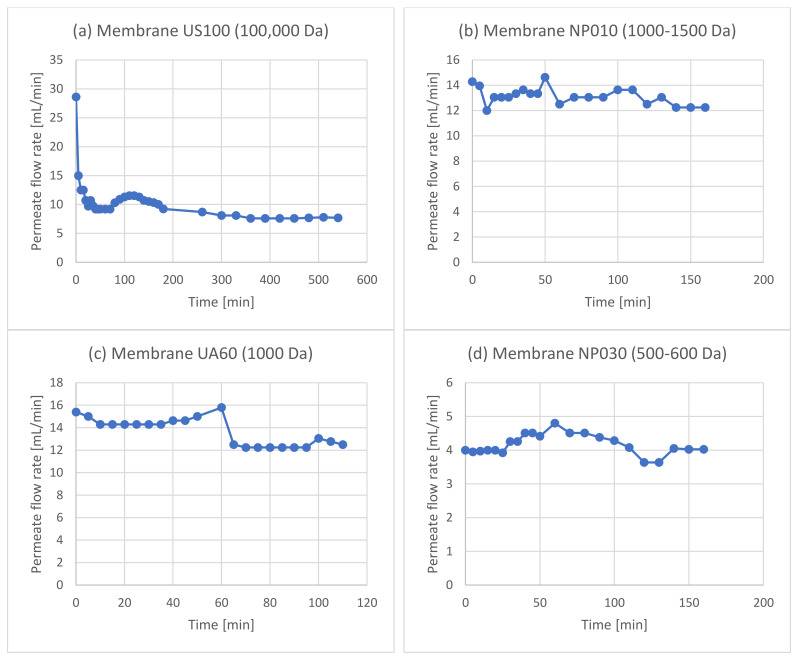
Permeate flow rates observed in the membrane treatment scheme after solid–liquid extraction in which only one ultrafiltration step ((**a**) US100) was used and the permeate from the 100,000 Da membrane was split into three equal parts and was treated with three different nanofiltration membranes ((**b**) NP010, (**c**) UA60, (**d**) NP030).

**Figure A3:**
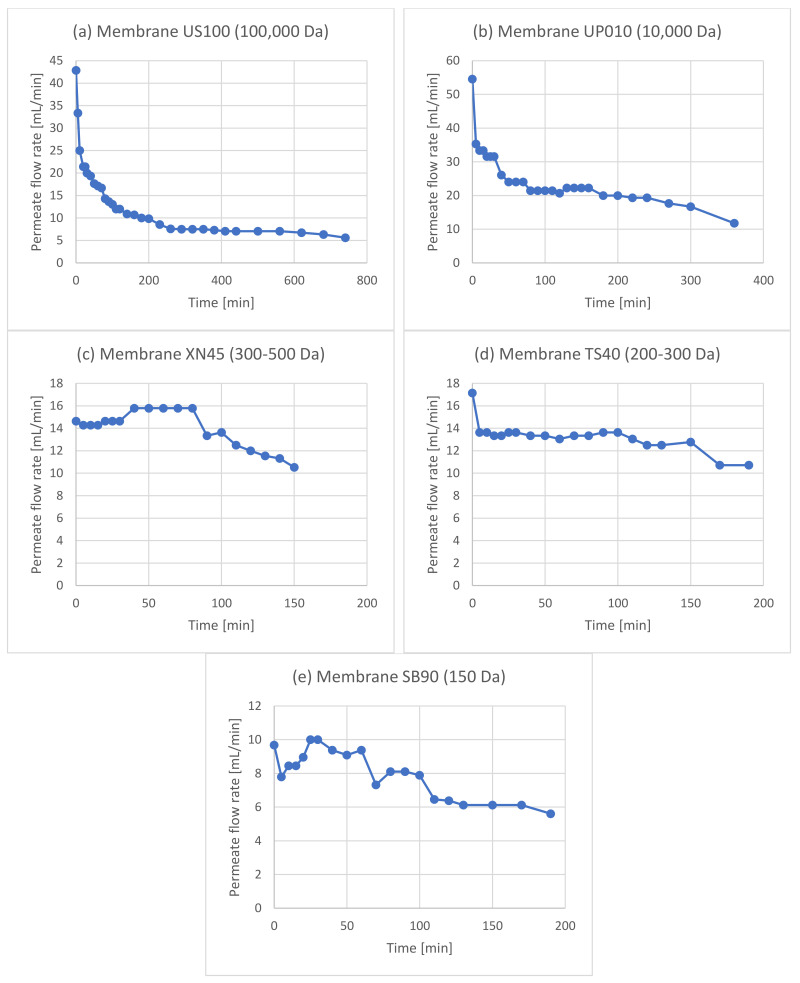
Permeate flow rates observed in the membrane treatment scheme after hydrolysis in which two ultrafiltration steps ((**a**) US100, (**b**) UP010) were used in line and the permeate from the 10,000 Da membrane was split into three equal parts and was treated with three different nanofiltration membranes ((**c**) XN45, (**d**) TS40, (**e**) SB90).

**Figure A4:**
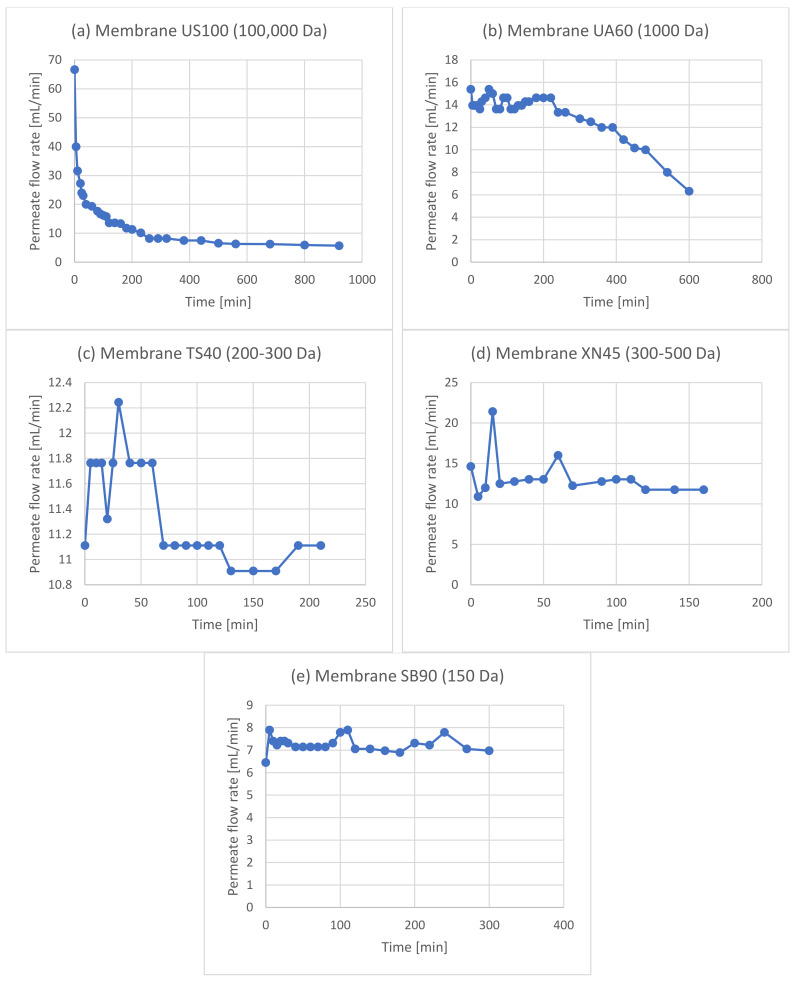
Permeate flow rates observed in the membrane treatment scheme after hydrolysis in which one ultrafiltration step ((**a**) US100) was used in line with a nanofiltration membrane ((**b**) UA60) and the permeate from the 1000 Da membrane was split into three equal parts and was treated with three different nanofiltration membranes ((**c**) XN45, (**d**) TS40, (**e**) SB90).

**Figure A5:**
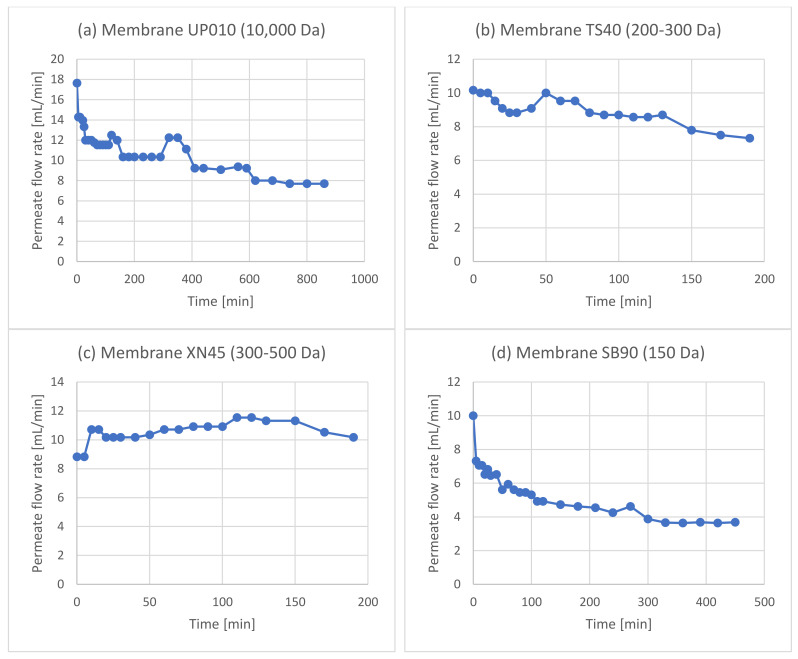
Permeate flow rates observed in the membrane treatment scheme after hydrolysis in which one ultrafiltration step ((**a**) UP010) was used and the permeate from the 10,000 Da membrane is split into three equal parts and was treated with three different nanofiltration membranes ((**b**) XN45, (**c**) TS40, (**d**) SB90).

The main goal of this work was to determine the suitability of membrane filtration for the enrichment of olive leaf extracts in hydroxytyrosol. In this scope, different membrane filtration schemes were tested in two different process steps. In two sets of experiments, membranes were used right after solid–liquid extraction (after ethanol recovery), and in three sets of experiments, membranes were used after hydrolysis of the extract with sulfuric acid. In each set of experiments, different membrane combinations were used in a decreasing MWCO order. All permeate flow rate and membrane rejection data are included in Appendix A (Figure A1, Figure A2, Figure A3, Figure A4 and Figure A5, Table A1).

### 3.1. Membrane Filtration after Solid–Liquid Extraction

**Figure 2:**
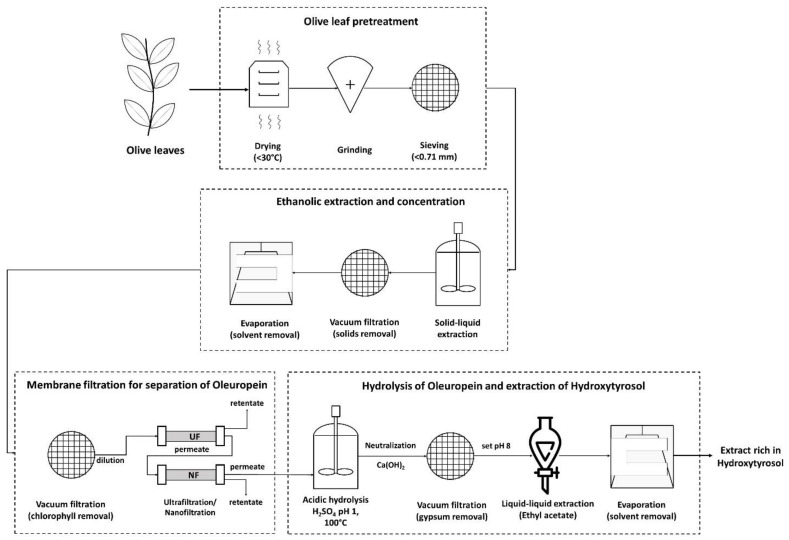
Process scheme in which the membrane filtration step takes place after the solid–liquid extraction.

As can be seen in Figure 2, this process scheme utilized the membrane filtration step after the solid–liquid extraction and more specifically, after evaporation of the solvent (ethanol) under vacuum. The concentration of ethanol was decreased below 5% for safety purposes and to avoid affecting the membrane performance due to swelling phenomena that can change membrane rejection and permeate flux [40,41]. Ethanol could also interfere with the liquid–liquid extraction step. Moreover, the recovery of ethanol to be reused in the extraction process can promote the environmental and economic sustainability of the proposed process, although this should be validated though a technoeconomic and life cycle assessment. After ethanol recovery, the evaporation residue was filtered to remove the solidified chlorophyll and other water-insoluble solids. The aqueous solution was then treated using the membrane filtration apparatus. This process scheme was tested via two membrane setups. The first setup used a 100,000 Da UF membrane (US100, Mann+Hummel, Ludwigsburg, Germany) to treat the initial liquid and a 10,000 Da UF membrane (UP010, Mann+Hummel, Ludwigsburg, Germany) to treat the permeate of the US100. The permeate of this membrane was divided into three equal parts and each one was treated with a different NF membrane (NP010, UA60, NP030, Mann+Hummel, Ludwigsburg, Germany). The second setup tested the same membranes as the first one but without the 10,000 Da UF membrane. These two experiments examine, firstly, the requirement of more than one UF step to ease the operation and to reduce fouling and concentration polarization issues, and secondly, the efficiency of each NF membrane to separate oleuropein from larger molecules.

To grasp the idea about the efficiency of the membranes, most of the permeates and retentates were analyzed for their concentrations in phenolics and sugars in GAE (mg/L) and glucose equivalents (mg/L), or their concentrations were correlated to the weight of the solid used for the solid–liquid extraction (g/kg dry leaves). However, the most significant factor that determined the best membrane sequence and the best process step to include was the recovery and purity of hydroxytyrosol in the final extract. As it was difficult to measure the concentrations of hydroxytyrosol and oleuropein in the complex extract solutions, they were extracted with ethyl acetate, evaporated to remove the organic solvent, and then they were redissolved in methanol to be analyzed, using HPLC. In the case of the first process scheme, it was necessary to hydrolyze the permeate from the NF membranes, because hydroxytyrosol quantification is much easier than oleuropein and because hydrolysis would be included in any case in the process since hydroxytyrosol is the target compound.

**Figure 3:**
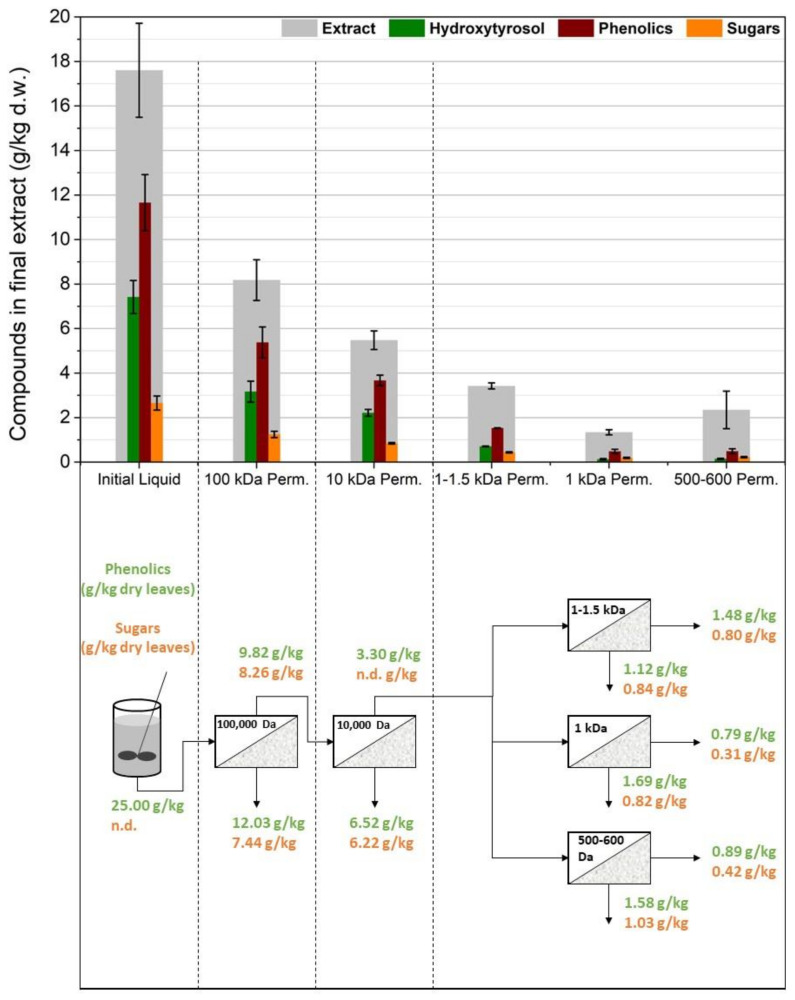
The first membrane treatment scheme: two ultrafiltration steps (US100, UP010) were used in line and the permeate from the 10,000 Da membrane was split into three equal parts and treated using three different nanofiltration membranes (NP010, UA60, NP030).

As shown in Figure 3, the 100,000 Da UF significantly reduced the concentration of oleuropein in the permeate (*p* = 0.049), therefore reducing hydroxytyrosol in the final extract. Even if oleuropein’s molecular weight was much lower than the MWCO of this membrane, it seems that fouling phenomena and some hydroxytyrosol that might be attached to larger molecules or particles led almost half of the available hydroxytyrosol into the retentate. A smaller reduction (*p* = 0.036) was also observed when the liquid was filtered using the 10,000 Da UF membrane. The permeates from the NF membranes exhibited significantly diminished concentrations of hydroxytyrosol (*p* = 0.012, 0.006, and 0.007 for the 1000–1500 Da, 1000 Da, and 500–600 Da membranes, respectively), showing a high oleuropein rejection. Out of the three NF membranes used, the 1000–1500 Da membrane (NP010), which was also the NF with the larger MWCO, was the most permeate for oleuropein.

**Figure 4:**
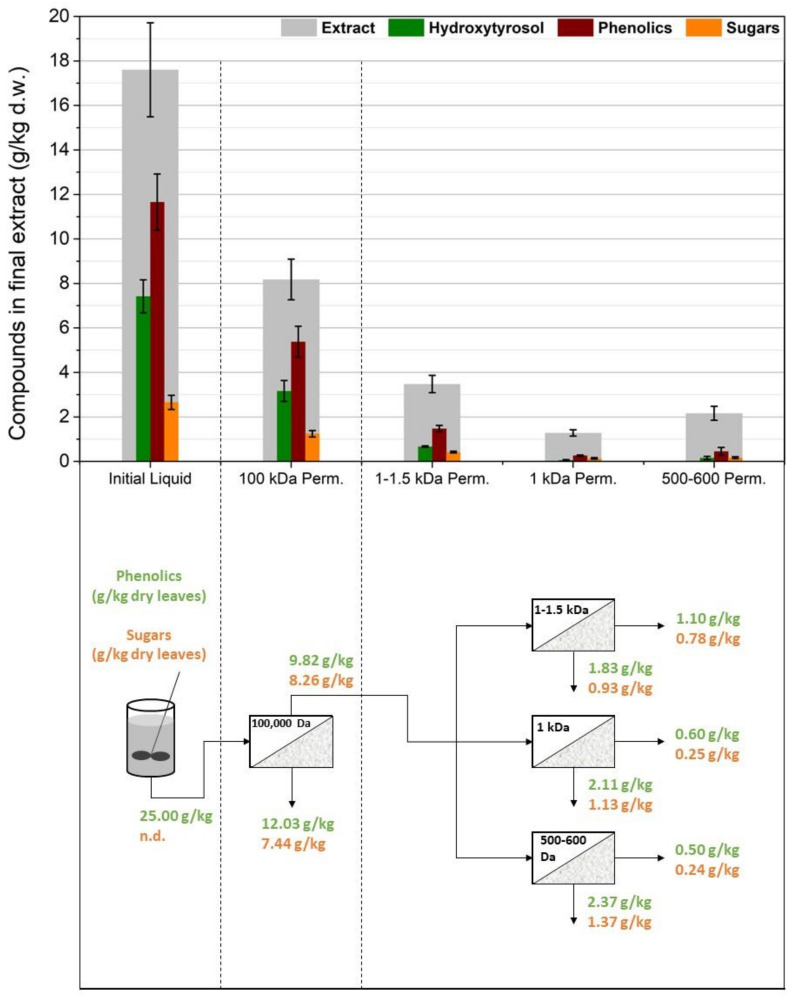
The second membrane treatment scheme: only one ultrafiltration step (US100) was used and the permeate from the 100,000 Da membrane was split into three equal parts and treated using three different nanofiltration membranes (NP010, UA60, NP030).

Figure 4 shows that, by removing the 10,000 Da membrane from the process, the amounts of phenolics, sugars, hydroxytyrosol, and total extract were approximately similar to the previous case (*p* > 0.05 for all three membranes in all the measured parameters). Therefore, the extra UF membrane does not improve the separation of oleuropein, and the fluxes during the operation of the NF membranes were similar to the previous experiment. Most solid particles and large macromolecules were removed by the 100,000 Da UF and it was adequate to avoid excessive fouling in the later NF membranes.

Overall, this process scheme did not give satisfactory results, and oleuropein tends to be rejected in the retentate despite its relatively small molecular size (540.5 g/mol). Moreover, process-wise, using the membranes after solid–liquid extraction and before acidic hydrolysis creates some flux issues, especially in the first stage where most of the fouling phenomena occur.

### 3.2. Membrane Filtration after Acid Hydrolysis

**Figure 5:**
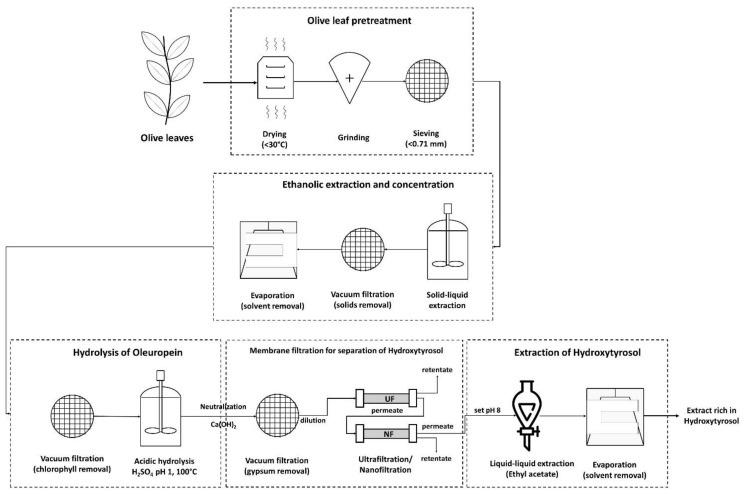
Process scheme in which the membrane filtration step took place after the acidic hydrolysis.

**Figure 6:**
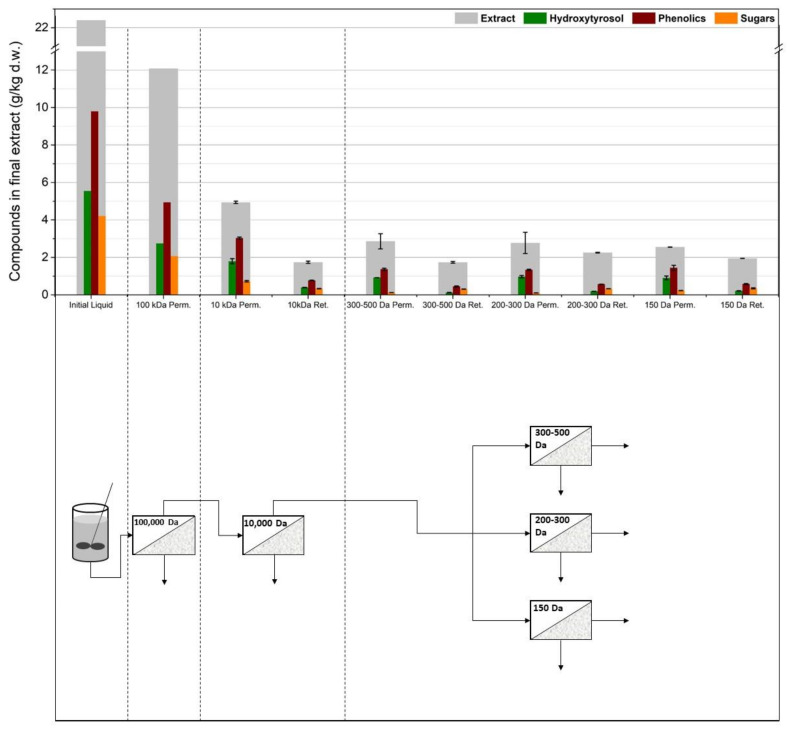
The first membrane treatment scheme: two ultrafiltration steps (US100, UP010) were used in line and the permeate from the 10,000 Da membrane was split into three equal parts and was treated with three different nanofiltration membranes (XN45, TS40, SB90).

**Figure 7:**
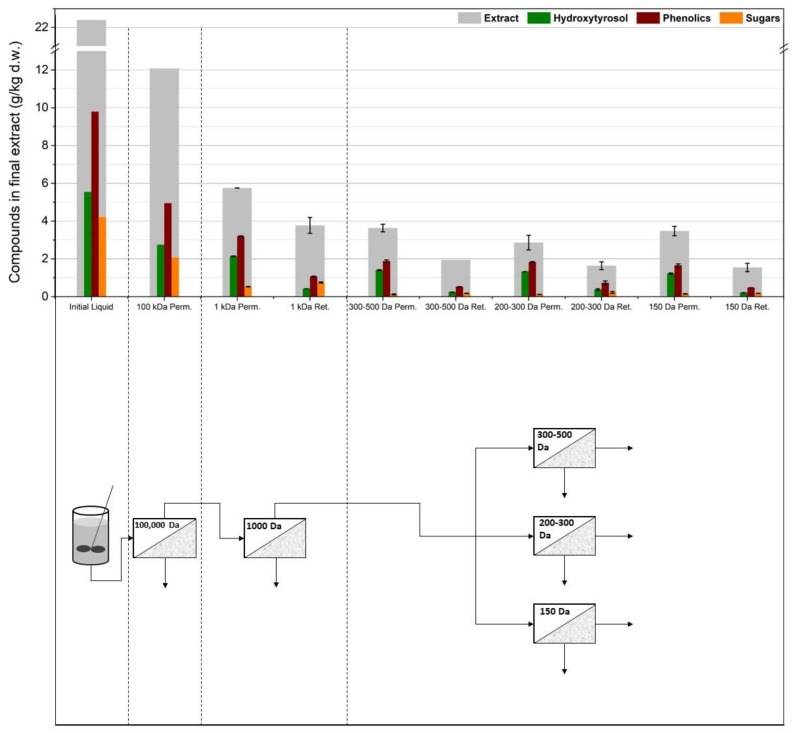
The second membrane treatment scheme: one ultrafiltration step (US100) was used in line with a nanofiltration membrane (UA60) and the permeate from the 1000 Da membrane was split into three equal parts and was treated with three different nanofiltration membranes (XN45, TS40, SB90).

**Figure 8:**
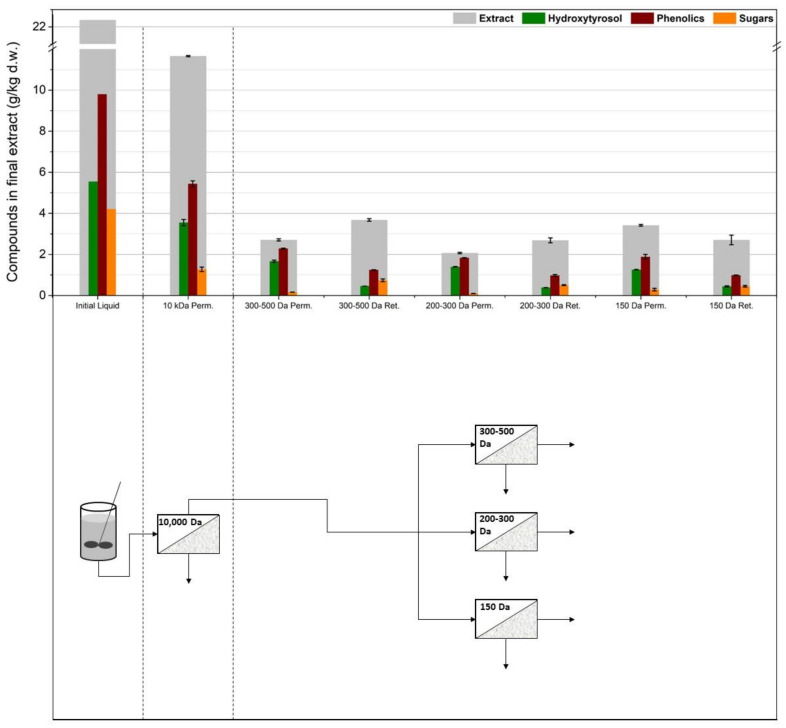
The third membrane treatment scheme: one ultrafiltration step (UP010) was used and the permeate from the 10,000 Da membrane was split into three equal parts and was treated with three different nanofiltration membranes (XN45, TS40, SB90).

As can be seen in Figure 5, the membranes were tested after acid hydrolysis of the extract, with the rationale that hydroxytyrosol would be easier to separate and that the hydrolyzed extract overall would cause less severe fouling phenomena due to smaller molecules in the solution and fewer particles present because of the extra filtration step after the neutralization of the hydrolyzed solution. To test this process scheme, as previously, different membrane setups were used to figure out the best configuration. The first (Figure 6) implemented two consecutive UF membrane steps (US100 and UP010, Mann+Hummel, Ludwigsburg, Germany) after the hydrolysis and filtering of the extract solution. The permeate from the 10,000 Da was again split into three equal portions, each to be treated with a different NF membrane (XN45, TS40, and SB90, Mann+Hummel, Ludwigsburg, Germany). The second setup (Figure 7) included a 1000 Da membrane after the 100,000 Da one, and the permeate from it was again split into three equal parts and was treated with the same NF membranes as in the previous setup. The third setup (Figure 8) did not use a 100,000 Da membrane at all and used only one 10,000 Da membrane (UP010), with its permeate again ending up in the same three NF membranes as in the other two cases. The NF membrane selection, in this case, was made based on hydroxytyrosol’s molecular weight (154 g/mol). As it can be seen in Figure 6, Figure 7 and Figure 8, the three NF permeates had very small differences in the amounts of hydroxytyrosol, phenolics, and sugars, with the 300–500 Da and the 200–300 Da having slightly larger recoveries of these substances than the 150 Da one but with no statistical significance (*p* = 0.64 and 0.51, respectively). However, the 150 Da membrane showed significant permeability in terms of hydroxytyrosol, despite having an MWCO very close to the MW of the molecule. Moreover, the extra UF steps did not play a significant role (apart from the case of the total phenols and HT measurement in the permeate of the 300–500 Da NF membrane, *p* = 0.029 and *p* = 0.048, respectively); on the contrary, they might reduce hydroxytyrosol in the final extract since more losses are ending up in the retentate at each membrane stage. This fact indicated that a “loose” UF membrane such as US100 would not be necessary to treat this liquid and, for that reason, the third setup treated the neutralized hydrolysate directly with the UP010 (10,000 Da). Operation with this membrane took place smoothly and no significant drops in the flux of the permeate were observed. This led to a UF permeate with slightly larger quantities in hydroxytyrosol and phenolics than the setups that used a 100,000 Da pretreatment step (*p* = 0.013) and, therefore, slightly larger quantities in the permeates of the NF membranes. The third setup had both higher recoveries and purity of phenolics and hydroxytyrosol and used fewer stages, which is very convenient process-wise. The highest recovery was achieved by the XN45 (300–500 Da), with 1.67 g/kg of dry leaves, while the highest purity of hydroxytyrosol as a weight percentage of the final extract was 68% with the TS40 (200–300 Da). In the other two setups, the recovery of hydroxytyrosol was slightly reduced and the purity of hydroxytyrosol hardly ever surpassed 40% *w*/*w*. 

## 4. Conclusions

Pressure-driven membrane separation processes can separate substances mainly based on their size and sometimes based on the interactions that take place between molecules or between molecules and surfaces (e.g., hydrophobic interactions in the membrane layer). Although membranes may be chosen only by their molecular weight cut-off, when chosen right they may effectively separate target compounds from a solution. When the process operates with the membrane retentate being recycled back to the feed, it becomes a feed concentration process, with molecules smaller than the MWCO along with the solvent ending up in the permeate stream. Membrane filtration was used to further improve the performance of a phenolic compound extraction technique previously studied by the authors. In the present study, it was shown that, when the membranes were used before acidic hydrolysis of an olive leaf extract, the overall process was hindered, especially at the first step with the UF membrane. This was attributed to the low permeate fluxes and excessive fouling phenomena, as well as significant losses in the amount of oleuropein ending up in the permeates and hydroxytyrosol being recovered in the final product. On the other hand, when membranes were used after acidic hydrolysis, the obtained permeate fluxes were not significantly impaired. Finally, the most efficient membrane setup was the in-line use of UP010 (10,000 Da) and TS40 (200–300 Da). This setup provided the highest fluxes and required only two membrane stages, instead of three included in other cases, and the highest recovery for hydroxytyrosol. TS40 resulted in hydroxytyrosol of greater purities compared with the other NF membranes, while fluxes were more stable and adequate. The increase in hydroxytyrosol purity in the final extract that was achieved through the membrane treatment was from approximately 25% *w*/*w* to 68%, with 25% being the purity achieved via the initial process, without the use of membranes. Considering that, in a previous work of the authors, a purity of 60% was achieved without the use of membranes, probably because of higher oleuropein concentrations in the olive leaves, there lies the potential of acquiring even higher hydroxytyrosol concentrations with the right feedstock and the process proposed in this work.

## Figures and Tables

**Table 1:** Characteristics of membranes used in this study.

Membrane	Membrane Type	Membrane Material	Permeability (LMH/bar)	MWCO (Da)
US100	UF	Polysulfone (PSUH)	>100	100,000
UP010	UF	Polyethersulfone (PES)	>50	10,000
NP010	NF	Polyethersulfone (PES)	>5	1000–1500
UA60	NF	Thin-Film Polypiperazine	76.5–136 (LMH)	1000
NP030	NF	Polyethersulfone (PES)	>1	500–600
XN45	NF	Thin-Film Polypiperazine	47.6–73.1 (LMH)	300–500
TS50	NF	Piperazine		300
TS40	NF	Thin-Film Polypiperazine	40.8–61.2 (LMH)	200–300
SB90	NF	Cellulose Acetate (CA)	44.2–69.7 (LMH)	150
X-20	RO	Thin-Film Polyamide	47.6–71.4 (LMH)	<150

## Data Availability

The data presented in this study are available on request from the corresponding author.

## Abbreviations

Cellulose acetate	CA
Gallic acid equivalents	GAE
Molecular weight cut-off	MWCO
Nanofiltration	NF
Polyethersulfone	PES
Polyvinylidene fluoride	PVDF
Polysulfone	PSUH
Trifluoroacetic acid	TFA
Ultrafiltration	UF

## Appendix A

**Table A1:** Apparent rejection of the membranes used in the experiment with two UF steps (1.1) and one UF step (1.2) prior to the NF membranes, prior to oleuropein hydrolysis and the experiments with two UF steps (2.1), one loose UF step (2.2), and one tight UF step (2.3) prior to NF, after oleuropein hydrolysis.

Experiment	Membrane	Rejection of Phenols	Rejection of Sugars
1.1	US100	54%	53%
	UP010	32%	32%
	NP010	58%	48%
	UA60	87%	77%
	NP030	87%	73%
1.2	US100	54%	53%
	NP010	73%	66%
	UA60	95%	89%
	NP030	92%	87%
2.1	US100	54%	53%
	UP010-P	44%	41%
	XN45-P	55%	83%
	TS40-P	56%	85%
	SB90-P	52%	68%
2.2	US100	50%	51%
	UA60	35%	74%
	XN45	41%	77%
	TS40	43%	78%
	SB90	48%	72%
2.3	UP010	44%	70%
	XN45	58%	87%
	TS40	66%	92%
	SB90	65%	77%
